# A strategy to reconstitute immunity without GVHD via adoptive allogeneic Tscm therapy

**DOI:** 10.3389/fimmu.2024.1367609

**Published:** 2024-07-05

**Authors:** Liping Guan, Yunqin Sun, Yanli Si, Qingya Yan, Ziyu Han, Youxun Liu, Tao Han

**Affiliations:** ^1^ School of Basic Medical Sciences, Xinxiang Medical University, Xinxiang, China; ^2^ Clinical Department, Sanquan College of Xinxiang Medical University, Xinxiang, China; ^3^ Xinxiang Key laboratory for Molecular Oncology, Institutes of Health Central Plains, Xinxiang Medical University, Xinxiang, China

**Keywords:** T memory stem cells, graft-versus-host disease, alloreactive T cells, adoptive immunotherapy, immune reconstitution

## Abstract

**Introduction:**

Adoption of allogeneic T cells directly supplements the number of T cells and rapidly induces T-cell immunity, which has good efficacy for treating some tumors and immunodeficiency diseases. However, poor adoptive T-cell engraftment and graft-versus-host disease (GVHD) limit the application of these methods. Alloreactive T-cell clones were eliminated from the donor T-cell repertoire, and the remaining T-cell clones were prepared as Tscm for T-cell adoptive treatment to reconstruct recipient T-cell immunity without GVHD.

**Methods:**

The subjects in this study included three different strains of mice. Lymphocytes from mice (C57BL/6) were used as the donor T-cell repertoire, from which the Tscm allo-reactive T cell clone was depleted (ATD-Tscm). This was confirmed by showing that the Tscm was not responsive to the alloantigen of the recipient (BALB/c). To prepare ATD-Tscm cells, we used recipient lymphocytes as a simulator, and coculture of mouse and recipient lymphocytes was carried out for 7 days. Sorting of non-proliferative cells ensured that the prepared Tscm cells were nonresponsive. The sorted lymphocytes underwent further expansion by treatment with TWS119 and cytokines for an additional 10 days, after which the number of ATD-Tscm cells increased. The prepared Tscm cells were transferred into recipient mice to observe immune reconstitution and GVHD incidence.

**Results:**

Our protocol began with the use of 1×10^7^ donor lymphocytes and resulted in 1 ×10^7^ ATD-Tscm cells after 17 days of preparation. The prepared ATD-Tscm cells exhibited a nonresponse upon restimulation of the recipient lymphocytes. Importantly, the prepared ATD-Tscm cells were able to bind long and reconstitute other T-cell subsets *in vivo*, effectively recognizing and answering the “foreign” antigen without causing GVHD after they were transferred into the recipients.

**Discussion:**

Our strategy was succeeded to prepare ATD-Tscm cells from the donor T-cell repertoire. The prepared ATD-Tscm cells were able to reconstitute the immune system and prevent GVHD after transferred to the recipients. This study provides a good reference for generating ATD-Tscm for T-cell adoptive immunotherapy.

## 1 Introduction

The innate and adaptive immune systems serve as the body’s defenses against infections. The innate immune system, which includes complement proteins and phagocytic cells such as monocytes, macrophages, and neutrophils, can combat intracellular infections. The adaptive immune system is primarily mediated by T and B lymphocytes, with T cells being the main effector cells that clear intracellular pathogen infections and tumor cells (1). Due to the destruction of tumors and the toxic side effects of radiotherapy and chemotherapy, the immune function of patients with malignant tumors is impaired (2, 3). A certain number of T lymphocytes in the body are an important cornerstone of various antitumor therapy methods (4). T-cell adoptive therapy (ACT) can directly increase the number of T cells, rapidly rebuild T-cell immune function, and subsequently promote anti-infection and antitumor effects (5–7). However, the aforementioned T-cell immune reconstitution has two major problems. After direct adoptive donor T cells reach the recipient, adoptive T cells exhibit poor engraftment and cannot survive long-term in the body or recapitulate T-cell immunity (8); on the other hand, graft-versus-host disease (GVHD) occurs following T-cell transfer and seriously affects the therapeutic efficacy of these cells (9).

T memory stem cells (Tscm) are a T-cell subset characterized by both memory T cells and stem cells (10–12). During the differentiation of Tn cells into various T-cell subsets, Tscm cells are the earliest differentiated T-cell subset. In the absence of antigen stimulation, the lifespan of initial lymphocytes is generally only a few months (13–15), while Tscm cells have strong self-renewal and differentiation capacities *in vivo* and can survive and self-renew without antigen stimulation, additionally, these cells can survive *in vivo* for decades after infusion (16–18). Thus, Tscm cells can be used as seed cells for allograft T-cell immune reconstitution.

GVHD occurs because T cells in the graft recognize the recipient alloantigen, and the rejection reaction is caused by launching an immune attack (19). The T cells in the donor T-cell bank, also called alloreactive T cells, can directly recognize the alloantigen and produce a response, which is the basis of GVHD, and this response is also associated with peptide-MHC complex (pMHC) (20, 21). The precursor frequency of alloreactive T cells is approximately 0.01 to 0.1 of the T-cell repertoire (22), and the vast majority of the remaining T-cell clones in the T-cell repertoire recognize “foreign” antigens and are the basis of immune reconstitution. Therefore, if the T-cell clones targeting the recipient alloantigen in the graft can be eliminated, the development of GVHD during immune reconstitution is expected to be avoided (23), and the retaining T-cell clones that recognize the “foreign” antigen is expected to prevent the occurrence of GVHD during immune reconstitution.

In our previous study, the same antigen-specific Tscm were successfully prepared from human peripheral blood T cells. The preparation method used included three steps (24): (a) Production of Tscm: stimulating differentiation of Tn cells into Tscm cells using an antigen or a CD3/CD28 antibody. (b) Enrichment of Tscm: intervention with inhibitors of differentiation (e.g., inhibitors of the Wnt-catenin signaling pathway TWS119) or transcription factor therapy and blockade of Tscm further differentiated into other subpopulations, thus increasing Tscm (25–27). (c) Expansion of Tscm cells: cytokines such as IL-7, IL-15 and IL-21 can proliferate Tscm cells and maintain their stem cell characteristics through the use of the cytokines IL-7 and IL-15, which are capable of expanding Tscm cells to a sufficient amount to meet experimental conditions (28, 29). In this study, we explored a methodology for preparing allo-reactive T-cell clone-depleted Tscm cells for adoptive treatment of T cells. More importantly, our findings demonstrated that allo-reactive T-cell clone-depleted Tscm cells can efficiency avoid GVHD and reestablish T-cell immunity.

## 2 Methods

### 2.1 Mice and antibodies

The subjects in this study included three different strains of mice. Six- to eight-week-old C57BL/6, BALB/c and C3H female mice were purchased from SPF (Beijing) Biotechnology Co., Ltd. Lymphocytes from C57BL/6 mice were used as the donor T-cell repertoire, BALB/c mice were used as the recipient, and another irrelevant mouse (C3H) was selected as the control for this study. All animal experiments were carried out according to protocols approved by the Animal Care Committee of Xinxiang Medical University and complied with the ARRIVE guidelines and carried out in accordance with the U.K. Animals (Scientific Procedures) Act, 1986 and associated guidelines. Fluorescent antibodies for cells staining included mouse CD3-PE (clone 17A2), CD8a-APC (clone 53–6.7), CD62L-PE (cloneW18021D), CD44-BV510 (clone IM7), CD122-BV421 (clone 5H4), H-2Kb-PerCP/Cyanine5.5 (clone AF6–88.5), IL-2-APC (clone 5H4), IFN-γ-APC (clone MOB-47), TNF-α-APC (clone MP6-XT22), CD107a-APC (clone 1D4B) the above antibodies were purchased from BioLegend, USA. The corresponding isotype for each antibody was used as an isotype control. The cells were analyzed on a Beckman CytoFLEX flow cytometer. T-cell subsets were evaluated using fluorescence minus one (FMO) as a control for antibodies of interest.

### 2.2 Isolation of splenocytes

Under sterile conditions, the mice were euthanized, and the spleens were removed from 6 cm sterile dishes with 5 ml of PBS. The 5 ml syringe piston was removed to grind the spleen into a suspension in a Petri dish, which was subsequently filtered through a 70 μm pore size filter. The suspension was transferred to a centrifuge tube at 1000 rpm for 10 min, after which 3 ml of red cell lysate was added to the cell precipitate. The mixture was gently blown for 1 min and washed twice with 10 ml of PBS. RPMI 1640 culture medium containing 10% FBS was added to the cell precipitate, which was subsequently added to a 6-well plate. After 6–10 h, the adherent cells were removed, and the nonadherent cells were cocultured with the cells.

### 2.3 Allogeneic coculture and ATD-Tscm preparation

Allogeneic lymphocytes (C57BL/6 and BALB/c) were cocultured to raise alloreactive T cells. Lymphocytes from C57BL/6 mice were stained with 5μΜ carboxyfluorescein diacetate succinimidyl ester (CFSE, Sigma) for 8 min at 37°C and washed with RPMI 1640 supplemented with 10% FBS three times. Lymphocytes from BALB/c mice were inactivated by irradiation (2.0 Gy). Lymphocytes from C57BL/6 and BALB/c mice were mixed on day 0 at a ratio of 5:1 for the first week. Lymphocytes from C57BL/6 mice cultured alone were used as controls. The medium was replaced on Day 4, and the cells were counted by trypan blue exclusion. As the allo-specific T cells proliferated in the coculture, the CFSE bright cells were sorted via by FACS with BD FACS Aria II on day 7. The sorted cells were stimulated with CD3/CD28 beads (10:1) in the presence of 5 μM TWS119 for 3 days and then expanded with the cytokines IL-7 and IL-15 (PeproTech, USA) at 25 ng/mL each for an additional week. T-cell subsets in the coculture bulks were identified by their surface markers: Tscm (H-2Kb+ CD3+ CD44 low CD62L high CD122 high) and naive T cells (CD44 low CD62L highCD122 low) and central memory T (Tcm) (CD44 high CD62L high) cells.

### 2.4 *In vitro* Tscm cell differentiation assay and intracellular cytokine staining

The prepared ATD-Tscm cells were incubated with lymphocytes from C57BL/6 mice, BALB/c mice, C3H mice or Dynabeads T-Activator CD3/CD28 (α-CD3/CD28) (Gibco, USA) at a ratio of 5:1. The cells were collected at 6 and 12 h of postincubation and labeled with the corresponding fluorescent antibodies for differentiation assays. ATD-Tscm cells incubated with the above stimuli in the presence of 1× BFA (eBioscience, USA) were collected for intracellular IFN-γ, IL-2, and TNF-α staining after 6 h of incubation. First dye the surface antigen(method as before), then fix the membrane breaking with fixed membrane breaking solution (BD, USA), add cytokine antibody to avoid light for 40 minutes. The samples were analyzed on a Beckman CytoFLEX. The data analysis was performed with FlowJo software version 10.0 (Tree Star).

### 2.5 Assessment of graft versus-leukemia/viruses

The prepared ATD-Tscm cells were incubated with lymphocytes from BALB/c mice, and A20 at a ratio of 5:1, (α-CD3/CD28) was used as a positive control stimulus. The cells were collected at 4 h of postincubation and labeled with the corresponding fluorescent antibodies for differentiation assays. ATD-Tscm cells incubated with the above stimuli in the presence of 1× BFA (eBioscience, USA) were collected for intracellular CD107a and granzyme (eBioscience, USA) staining after 4 h of incubation. The samples were analyzed on a Beckman CytoFLEX. The data analysis was performed with FlowJo software version 10.0 (Tree Star).

The same method prepared human peripheral blood-derived ATD-Tscm, Inactivation of peripheral blood lymphocytes from healthy individuals were taken as stimulating cells, Lymphocytes from different individuals were taken as effector cells and co-culture with the stimulated cells. The same method as for the mouse ATD-Tscm preparation. In humans, the phenotypic markers of Tscm are CD45RA+, CCR7+, CD62L+, CD95+. Then, the enzyme Enzyme-linked immunospot assay was used to detect CMV antigen-specific T cells. CMV PepPool, and 2 µg/ml of each peptide (Mabtech, Sweden) was added to 2.5×10^5^/100 µl ATD-Tscm and incubated in 96-well plates (Mabtech, Sweden) of Elispot IFN-γ for 16h. CMV PepPool, 2 µg/ml of each peptide (Mabtech, Sweden) was added to 2.5×10^5^/100 µl PBMC served as Tn control for, 1×10^5^/100μl PBMC with 2.5µg/ml PHA as positive control, 100μl of the cell culture medium containing 2.5×10^5^/100 µl ATD-Tscm served as a background control. The plates were washed according to the operating instructions, colored, dried and counted by enzyme-linked immunospot analyzer (AIDispot 08, Germany) after 16 h.

### 2.6 Assessment of T cell immune reconstitution and GVHD *in vivo*

Six-week-old, pretreated female mice were generated as follows: recipient and control mice were subjected to total body irradiation via an X-ray linear accelerator (total dose of 2.0 Gy, dose rate of 0.5 Gy/min); then, the recipient mice (BALB/c) and the irrelevant mice (C3H) (n=5) were injected with 8×10^6^ ATD-Tscm 1 day after irradiation. The mental status, physical signs, and survival period of the mice were monitored daily after ATD-Tscm-related transfer. Blood samples were taken every week from the mice after ATD-Tscm-related transfer. The number and phenotype of H-2Kb+ T cells in mouse peripheral blood were determined by flow cytometry. The serum concentrations of the cytokines IL-2, IFN-γ and IL-6 were measured via ELISA(R&D Systems, USA), 20 µl of tail vein blood serum were taken and strictly follow the operating instructions. Measured the absorbance at 450 nm using an enzyme-linked immunosorbent assay (ELISA) reader.

Mice were euthanized after ATD-Tscm-cell transfer for 42 days. Tissues such as the liver and small intestine were obtained for HE staining, and inflammation and necrosis were analyzed pathologically. After single blindness of intestinal and liver tissue sections, they were submitted to a professional animal pathologist and scored for mouse intestinal and liver acute GVHD (aGVHD) scores with reference to aGVHD clinical pathology grade (30, 31). The 0 score is considered as normal, 1 score shows a mild inflammatory cell infiltration, 2 score shows inflammatory infiltrates occurred in less than 20% areas, 3 score shows inflammatory infiltrates occurred in 20%-33.3% areas, 4 score shows inflammatory infiltrates occurred in 33.3%-50% areas, 5 score shows inflammatory infiltration occurred in greater than 50% areas, The score of the two organs is added up as the overall score.

### 2.7 Establishment of a mouse skin grafting model

Six-week-old BABL/c mice were treated as recipients and were randomly divided into 3 groups: transplanted recipient (BALB/c) mouse skin group, transplanted donor (C57BL/6) mice skin group and transplanted irrelevant (C3H) mice skin group (n=5). The back skin of the donor, recipient and irrelevant mice was removed and transplanted into the back of the recipient mice one week after adoptive ATD-Tscm. After euthanizing the donor mice, the whole skin on the back was cut into a 5 mm 6 mm skin sheet. Donor mice were anesthetized after conventional depilation in the back graft area and disinfected with skin cutting to make the skin grafting bed base. The graft fixed bands were removed 3d after transplantation and the graft patches were observed daily for 42 d.

### 2.8 Statistical analysis

The statistical significance of differences between two groups was assessed with a 2-tailed paired or unpaired t test. Comparisons of more than two groups were performed by one-way ANOVA with multiple comparison tests. The data are shown as the mean ± standard deviation (SD). Differences are marked as no significance (NS), P > 0.05; *P < 0.05; **P < 0.01; and ***P < 0.001. All the data obtained from the study were analyzed using SPSS 22.0 (IBM, USA).

## 3 Results

### 3.1 Preparation methods for ATD-Tscm cells

**Figure 1:**
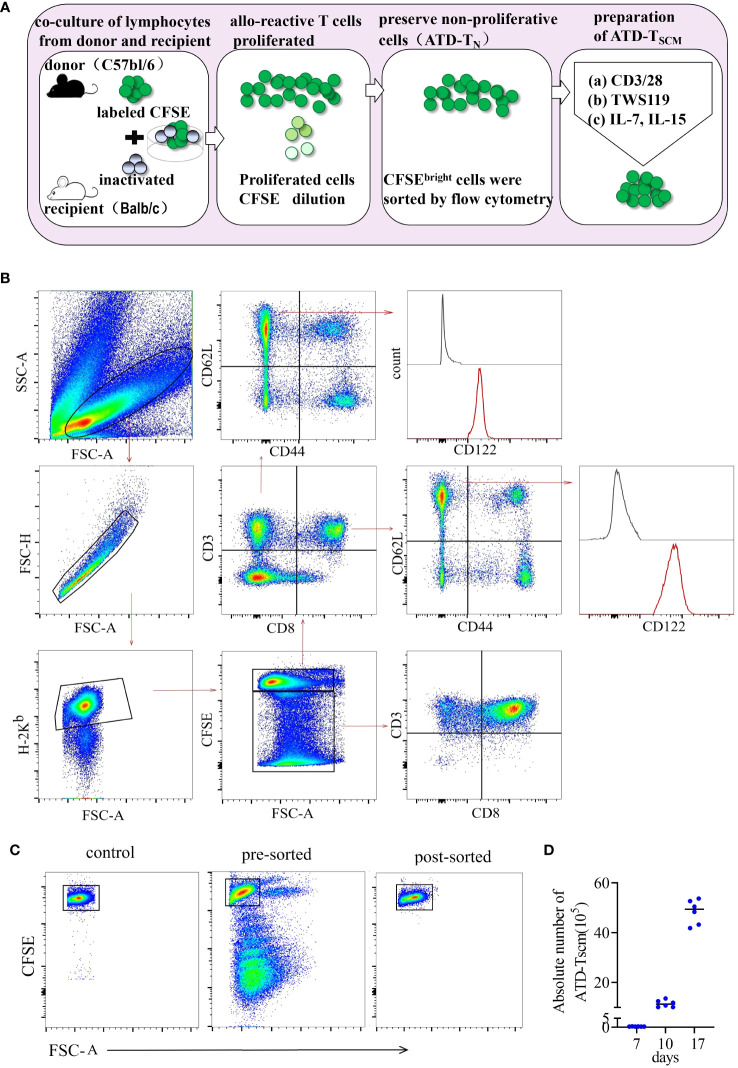
ATD-Tscm cells were effectively prepared *in vitro*. **(A)** Protocol for preparation of ATD-Tscm cells. The method began with setting up an alloreactive coculture by mixing C57BL/6 splenocytes and BALB/c allogeneic splenocytes on day 0 (allogeneic activation) to generate allospecific T cells. The CFSEbright cells in the coculture bulks were sorted by FACS on day 7 (nonproliferation sorting). The sorted cells were stimulated with beads coated with a CD3/CD28 antibody and 5 µM/L TWS119 for an additional 3 days (differentiation inhibition) and cultured with IL-7 and IL-15 (25 ng/mL each) to expand the Tscm for the next 7 days (cytokine expansion). The cultured bulks were subsequently used as the prepared Tscm cells for the *in vitro* and *in vivo* tests included in this study. **(B)** Gating strategy for identification of the H-2Kb+ CD3+ Tscm subset in the coculture bulks. Lymphocytes were identified based on their SSC versus FSC, and proliferating cells were identified by a reduced fluorescence intensity of CFSE (CFSEdim). Among the gated CFSEbright population, H-2Kb+ and CD3+ cells were selected for further CD44 and CD62L analysis. The Tscms exhibited the phenotype of CD3+ CD44low CD62L+CD122+. The illustration is representative of coculture bulks analyzed by FCM on day 7 before sorting, and the white peaks in the CD122 plots are isotype controls. The broken arrows indicate the sequential gating strategy. **(C)** ATD-Tn cells maintain nonproliferation in coculture, and CFSEbright cells were sorted by FACS with a purity above 98%. **(D)** Tscm numbers increased 150-fold in the coculture bulks with TWS119 enrichment and cytokine expansion after 10 days. The data are presented as the mean ± SD of six individual experiments.

To meticulously prepare ATD-Tscm cells, we first meticulously designed the procedure. The protocol for the preparation of ATD-Tscm cells is depicted in **Figure 1A**. We utilized inactivated recipient (BALB/c) spleen lymphocytes as the stimulators and donor (C57BL/6) spleen lymphocytes, labeled with CFSE, as the responders. These two types of lymphocytes were cocultured at a ratio of 1:5 (stimulating cells to reactive cells), and the cells were harvested on day 7. Within the coculture system, Tscm cells were identified by their phenotype: H-2Kb+ CD3+ CD44low CD62Lhigh CD122high (as shown in **Figure 1B**). Allo-specific T cells from the donor lymphocytes underwent proliferation, which resulted in a decrease in CFSE fluorescence. Based on the CFSE fluorescence intensity, proliferating lymphocytes were eliminated using flow cytometry (**Figure 1C**), and the viable cells that did not proliferate were collected, namely, Tn cells from which alloreactive T-cell clones had been removed (designated as ATD-Tn). The ATD-Tn cells were then collected and cocultivated with CD3/CD28 antibody-coated beads at a ratio of 10:1. TWS119 (5 μM/L) was added to prevent further differentiation of Tscm cells for a period of 3 days. This inhibition of differentiation resulted in a 50-fold increase in the number of Tscm cells by day 10. Additionally, the supplementation of IL-7 and IL-15 at concentrations of 25 ng/mL for 7 days led to an approximately 150-fold expansion of Tscm cells compared to the sorted cells by day 17 (**Figure 1D**). To ascertain that the nonproliferating lymphocytes were indeed specifically unresponsive to the recipient’s alloantigens, we sorted the CFSEbright lymphocytes and cocultured them with both inactivated lymphocytes from the recipient and from irrelevant mice. After one week, the alterations in CFSE fluorescence were measured using flow cytometry. Our findings revealed that the sorted cells failed to proliferate upon stimulation with recipient lymphocytes (**Supplementary Figure S1**). This outcome confirmed that the method employed effectively eliminated alloreactive T cells.

### 3.2 The prepared ATD-Tscms exhibited no response to the alloantigen *in vitro*

**Figure 2:**
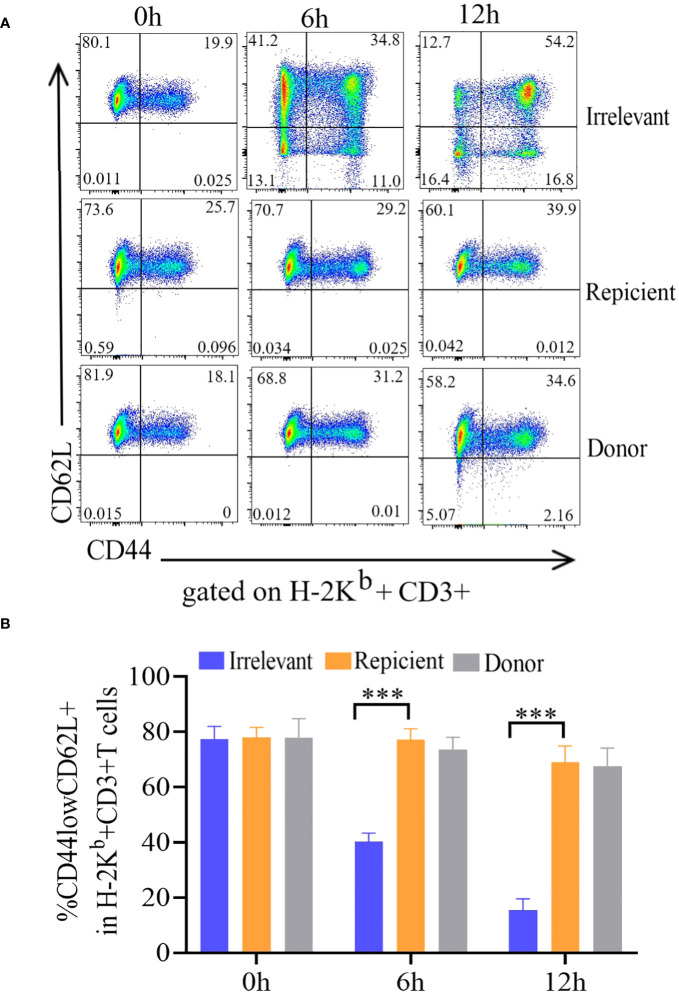
The ATD-Tscm cells did not respond to stimulation by recipient mouse lymphocytes. The prepared ATD-Tscms were stimulated with lymphocytes from irrelevant mice, recipient mice, or donor mice. The cell ratio was 5:1 at 0, 6 and 12 hours, and the frequency of T-cell subsets was determined by flow cytometry. ATD-Tscm cells differentiated into effector T cells upon stimulation with irrelevant factors. ATD-Tscm cells did not respond to recipient mouse or donor mouse lymphocytes. H-2Kb+ CD3+ T cells expressing CD44low and CD62L+ (ATD-Tscm cells), CD44high and CD62L+ (Tcm cells), CD44low and CD62L- (Tem cells) and CD44low and CD62L- (Tef cells) were detected at the indicated hours after stimulation. **(A)** Representative FCM plots are shown. **(B)** The data are presented as the mean ± SD of four individual experiments (n=4). ***P < 0.001..

To evaluate the nonresponse of the prepared ATD-Tscm cells to the recipient mouse alloantigen, the ATD-Tscm cells were cocultured with recipient, irrelevant and donor mouse lymphocytes to observe the ATD-Tscm response. The phenotype and frequency of each subset of ATD-Tscm cells and their differentiated T-cell subsets were determined by flow cytometry. The frequency of ATD-Tscm cells remained basically unchanged with recipient lymphocyte stimulation, and the frequency of other subsets of differentiated T cells was low (**Figure 2A**), basically no difference from that of the negative control (**Figure 2B**). After coculture with irrelevant mouse lymphocytes, ATD-Tscm differentiated into other subsets of T cells within 6 hours.

The immune effector functions mediated by T-cells encompass a range of activities, notably the production of cytokines and the elimination of target cells. To investigate cytokine production, Tscm cells were stimulated with recipient mouse lymphocytes, irrelevant mouse lymphocytes, or α-CD3/CD28 beads, after which the production of intracellular IL-2, TNF-α, and IFN-γ was measured.The irrelevant mouse lymphocyte-restimulated Tscm cells were more positive for these cytokines.

**Figure 3:**
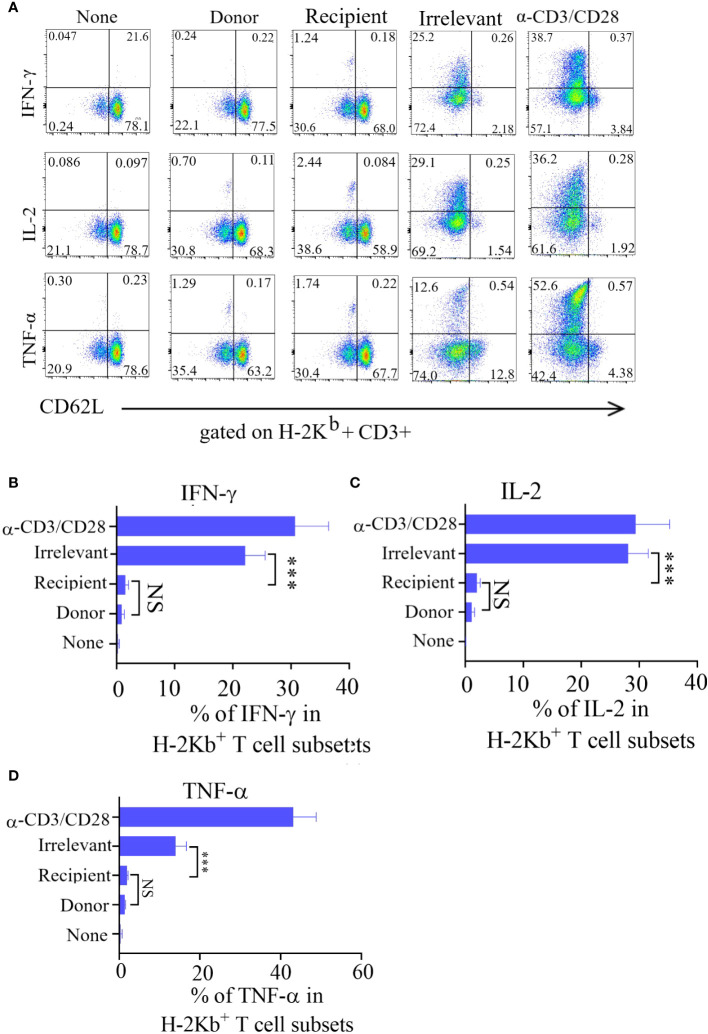
The ATD-Tscm did not secrete cytokines under the stimulation of recipient mouse lymphocytes. ATD-Tscm were cocultured with recipient lymphocytes, irrelevant mice or donor mice at a numerical ratio of 5:1 at 6 hours, after which the secretion of the cytokines IFN-γ, IL-2, and TNF-α by subsets of effector cells was examined via intracellular cytokine staining. **(A)** Representative FCM plots are shown. **(B–D)** The data are presented as the mean ± SD of four individual experiments (n=4). ***P < 0.001, NS: no significance P > 0.05.

After being cultured in combination with CD62L, the incubated bulks were divided into CD62L+ (Tscm and Tcm) and CD62L- (Tem and Tef) cells, and most of the cytokine-positive cells were Tem and Tef cells (**Figure 3A**). The results showed that the frequency of cytokine-positive Tscm cells was the same in mice stimulated with recipient lymphocytes and in mice not stimulated with these cells. In contrast, the Tscm cells responded to α-CD3/CD28 in a similar profile of cytokine production to that of lymphocytes from irrelevant mice (**Figures 3B–D**). These results indicated that the prepared ATD-Tscm cells did not respond to the alloantigen in the recipient mice.

### 3.3 The prepared ATD-Tscms exhibited the characteristics of T cell repertoire

**Figure 4:**
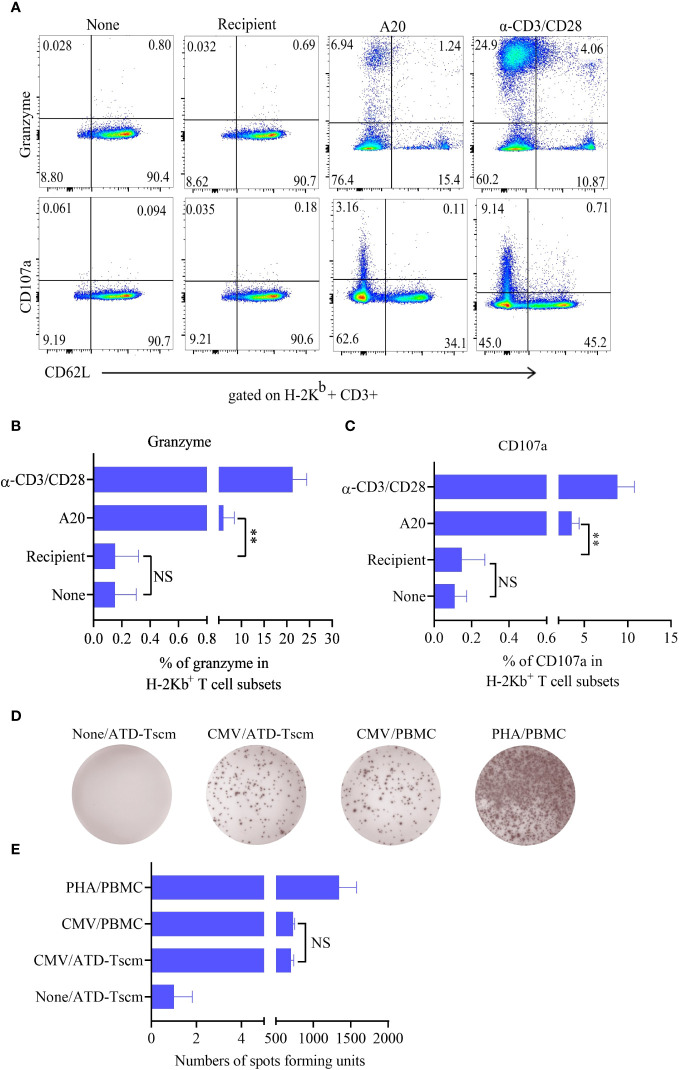
The ATD-Tscm can exert both anti-graft-versus-leukemia (GVL) and antiviral effects. The prepared ATD-Tscm cells were incubated with lymphocytes from BALB/c mice, and A20 at a ratio of 5:1, (α-CD3/CD28) was used as a positive control stimulus. The cells were collected at 4 h of postincubation and CD107a and granzyme were examined by FCM. **(A)** Representative FCM plots are shown. **(B, C)** The data are presented as the mean ± SD of four individual experiments (n=4). **(D)** Human-derived ATD-Tscm and Tn were stimulated with CMV PepPool, and the production of IFN-γ was assessed using Elispot. Representative Elispot images of IFN-γ spots. **(E)** Data analysis of spot counts per group showed no difference in the number of spots between ATD-Tscm and Tn upon CMV PepPool stimulation (n=4). NS, P > 0.05; **P < 0.01.

To test whether the prepared ATD-Tscm has the characteristics of a T cell repertoire i. e., which can recognize a “foreign antigen”, we examined anti-leukemia and anti-infection effects of the prepared ATD-Tscm. The ATD-Tscm cells were cocultured with recipient lymphocytes and A20 to observe the ATD-Tscm response. The frequency of CD107a and granzyme-positive cells, each subset of ATD-Tscm cells were determined by flow cytometry (**Figure 4A**). A20-stimulated Tscm cells were more positive for CD107a and granzyme than that of recipient lymphocytes-stimulated Tscm cells (**Figures 4B, C**). We then tested the precursor frequency of cytomegalovirus (MCMV) viral peptide-specific T cells and in Tn and ATD-Tscm, and compared the precursor frequency of peptide-specific T cells from both MCMV viruses to see whether the prepared ATD-Tscm was characteristic of a T cell repertoire. The results of Elispot IFN-γ showed that there were no or few spots in the blank control wells, and more spots should appear in the positive control wells.

The number of spots in the Tn and ATD-Tscm wells was between the blank control and the positive control (**Figure 4D**), and there was no difference in the number of spots between Tn and ATD-Tscm (**Figure 4E**). The above results suggest that the prepared ATD-Tscm has the ability to fight leukemia and viral infection i. e. has the characteristics of T cell repertoire.

### 3.4 The prepared ATD-Tscm cells are able to reconstruct T-cell immunity *in vivo*

**Figure 5:**
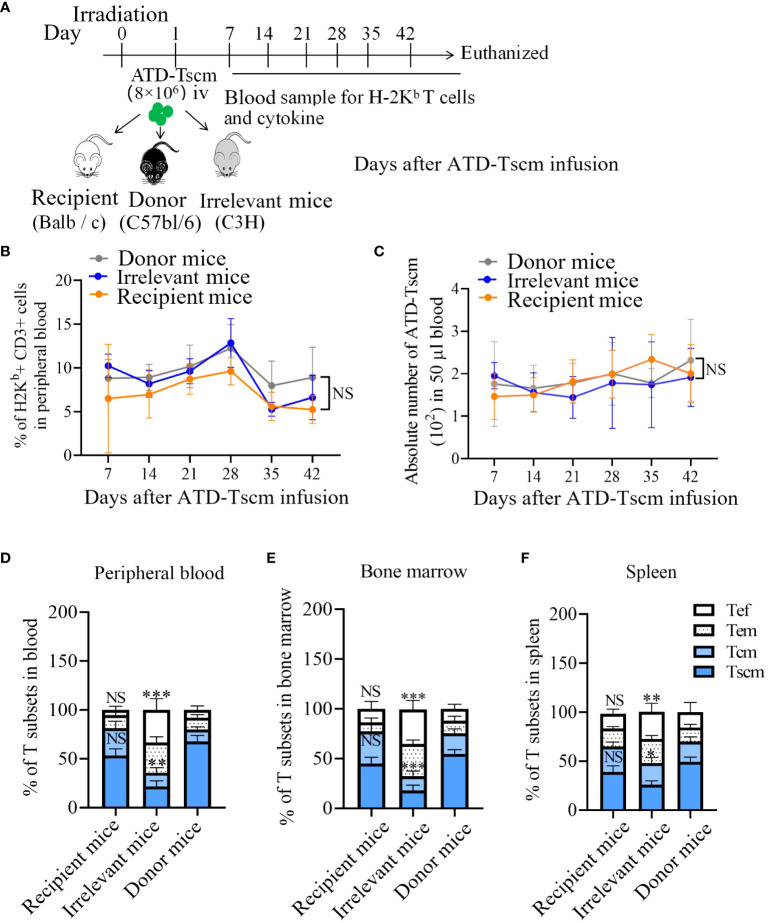
ATD-Tscm cells reconstructed T-cell immunity in mice. **(A)** Adoptive transfer protocol for ATD-TSCM. On day 1, ATD-Tscm cells were intravenously (iv) injected into the irradiated mice. Blood samples were taken once a week for 6 weeks after T-cell treatment. On day 42, the mice were sacrificed for detection of H-2Kb+ T cells. **(B)** The prepared ATD-Tscm cells were persistent *in vivo*. **(C)** The frequency of H-2Kb+ CD3+ T cells and the absolute number of ATD-Tscm cells in 50 μl of blood were detected via FCM. **(D–F)** The distribution of H-2Kb+ CD3+ T-cell subsets (Tscm, Tcm, Tem, and Tef) in the peripheral blood, spleen, and bone marrow was detected by FCM. More memory T-cell subsets (Tscm and Tcm) were detected in the donor and recipient mice. The data are presented as the means ± SD of five mice. NS, P > 0.05; *P < 0.05; **P < 0.01; and ***P < 0.001.

To observe T-cell immune reconstitution, recipient mice (BALB/c) and irrelevant mice (C3H) were treated with 8×10^6^ ATD-Tscm cells. On 7, 14, 21, 28, 35 and 42 days after T-cell infusion, caudal vein peripheral blood samples were taken to determine the frequency and phenotype of H-2K^b+^ T cells (**Figure 5A**). We found H-2Kb+ CD3+ T cells in the peripheral blood of ATD-Tscm-treated mice at all sampling times (**Figure 5B**). In addition, no difference was found in the absolute number of Tscm cells among the three groups of mice (**Figure 5C**).

To investigate the immune reconstitution capacity of the Tscm cells, the peripheral blood, spleen, and bone marrow of ATD-Tscm-treated mice were tested for H-2K^b+^ T cells on day 42. The H-2K^b+^ T cells in the above specimens consisted of ATD-Tscm cells (CD44low and CD62L+), Tcm cells (CD44high and CD62L+), Tem cells (CD44low and CD62L-) and Tef cells (CD44- CD62L-). The distribution of T-cell subsets revealed more Tem and Tef cells in the irrelevant mice than in the recipient and donor mice, whereas more Tscm and Tcm cells were found in the recipient and donor mice (**Figures 5D–F**). As Tem and Tef cells are at the terminal differentiation stage and Tscm and Tcm cells are at the early differentiation stage, our findings suggested that ATD-Tscm cells differentiate into other T-cell subsets when they cross the tissue antigens of irrelevant mice. These findings indicated that the ATD-Tscm cells were able to survive long through self-renewal in all the mice but did not recognize the alloantigen in the recipient mice and differentiated into other T-cell subsets when stimulated by “foreign” antigens to produce other subgroups in the irrelevant mice.

### 3.5 The prepared ATD-Tscm cells did not trigger GVHD *in vivo*

**Figure 6:**
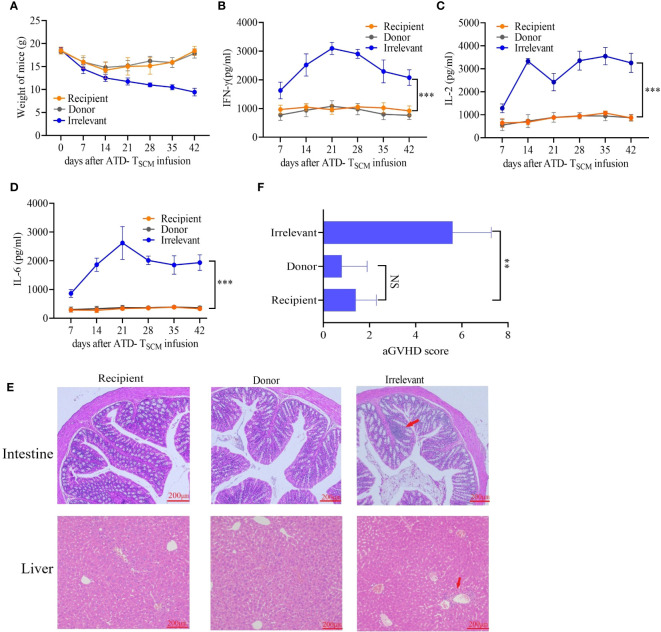
GVHD was detected in mice after ATD-Tscm-cell transfer. **(A)** Changes in the body weight of the mice in each group. **(B–D)** Serum IFN-γ, IL-2 and IL-6 concentrations in mice after adoptive ATD-Tscm therapy. The serum cytokine content in the recipient mice was not different from that in the donor mice at any time point. **(E)** Development of GVHD in mouse gut and liver tissues. The liver and intestine of the irrelevant mice exhibited lymphocyte infiltration (red arrow), but there were no significant changes in the recipient or donor mice. **(F)** GVHD scores were assigned based on the degree of inflammation, and the results showed no difference between the donor and recipient mice. NS, P > 0.05; **P < 0.01; and ***P < 0.001.

To monitor the occurrence of GVHD, the mice were closely observed daily for changes in mental status, body weight, and survival duration after the infusion of ATD-Tscm cells. Furthermore, the levels of cytokines IL-2, IFN-γ, and IL-6 in the bloodstream were measured using the enzyme-linked immunosorbent assay (ELISA) technique. Our meticulous observations indicated that all irradiated mice exhibited similar clinical manifestations during the first week following irradiation, marked by a progressive loss of body weight and a range of symptoms including lethargy, piloerection, and a hunched posture. Following this period, recipient mice that received the ATD-Tscm treatment started to show a gradual recovery in body weight. In contrast, the mice that were treated with lymphocytes from irrelevant sources continued to experience weight loss, culminating in death (**Figure 6A**). There were no differences in the levels of IL-2, IFN-γ, or IL-6 between recipient and donor mice. However, the levels of the cytokines described above increased in the irrelevant mice (**Figures 6B–D**). Upon euthanizing the mice on day 42, the small intestine and liver tissues from each group were harvested for hematoxylin and eosin (H&E) staining. Notable lymphocyte infiltration, epithelial cell necrosis in the intestinal tissue, and lymphocytic infiltration accompanied by swelling and necrosis in the liver’s vascular regions were observed in the irrelevant mice. In contrast, the organs of the recipient and control mice showed no significant pathological changes (**Figure 6E**). GVHD scores were assigned based on the degree of inflammation, and the results showed that the irrelevant mice had the highest GVHD scores, while there was no difference in scores between the recipient and donor mice (**Figure 6F**). These results indicate that GVHD does not develop after ATD-Tscm cells.

### 3.6 The prepared ATD-Tscms could exert T-cell function *in vivo*

**Figure 7:**
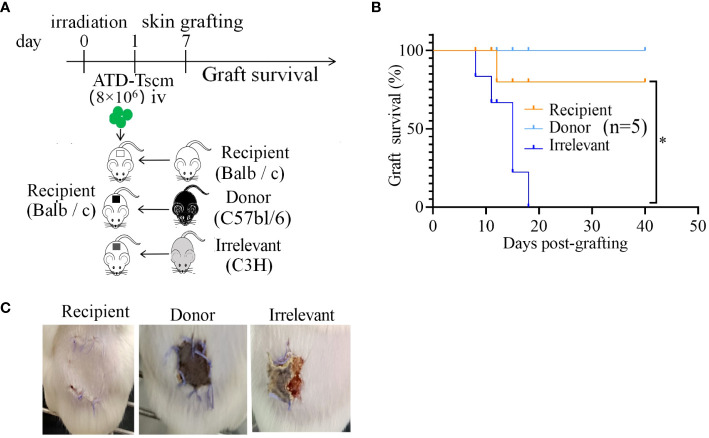
The skin grafts of irrelevant mice were removed from the recipients via ATD-Tscm-mediated transfer. **(A)** Adoptive ATD-Tscm mice were transplanted into the skin of donor, recipient and irrelevant mice. **(B)** Survival curve of the skin graft (n = 5). *P < 0.05; Mann−Whitney test. **(C)** The appearance of skin grafts of different origins in recipient mice at 12 days after transplantation.

Following the adoptive transfer of ATD-Tscm cells, we employed a mouse dorsal-to-dorsal skin transplantation model to assess the functional restoration of the recipient’s T cells. The experimental procedure is illustrated in **Figure 7A**. Initially, we infused 8×10^6^ ATD-Tscm cells into the recipient. Subsequently, after a period of seven days, the dorsal skin from both the irrelevant mice, donor mice and the recipient mice was transplanted onto the recipient mice’s backs. We noted that the skin grafts were rejected within a span of 20 days, and it is particularly noteworthy that recipient mice with grafts from irrelevant donors faced a complete rejection. In contrast, the recipient mice that received skin grafts from the original donors exhibited significantly enhanced survival of the grafts compared to those that received the irrelevant grafts (**Figure 7B**). Recipient mice that received skin grafts from irrelevant mice experienced complete graft loss, and the grafts were rejected by the recipient mice on day 12 post-transplantation. However, in recipient mice that underwent transplantation with skin from the donor and recipient mice, the grafts remained intact without edema, crusting, constriction, or detachment, indicating that the recipient mice accepted the skin grafts without any apparent signs of rejection (**Figure 7C**). In summary, recipient mice equipped with ATD-Tscm cells do not exhibit rejection of skin from transplanted donor mice, yet they do reject skin from irrelevant mice. This indicates that ATD-Tscm cells are capable of functioning within the recipient mice without inducing GVHD.

## 4 Discussion

During the process of thymic development, each T cell clone expresses a unique T cell receptor (TCR), each recognizing a specific antigenic epitope, that is, the antigen peptide-major histocompatibility complex (pMHC). The individual T cell repertoire represents the sum of various antigen-specific T cell clones within the body. Due to the negative selection in the thymus that removes T cell clones recognizing self-pMHC, the multitude of T cell clones within the body endows the organism with the ability to recognize a variety of “foreign” antigens from the natural world and to generate responses (32).

**Figure 8:**
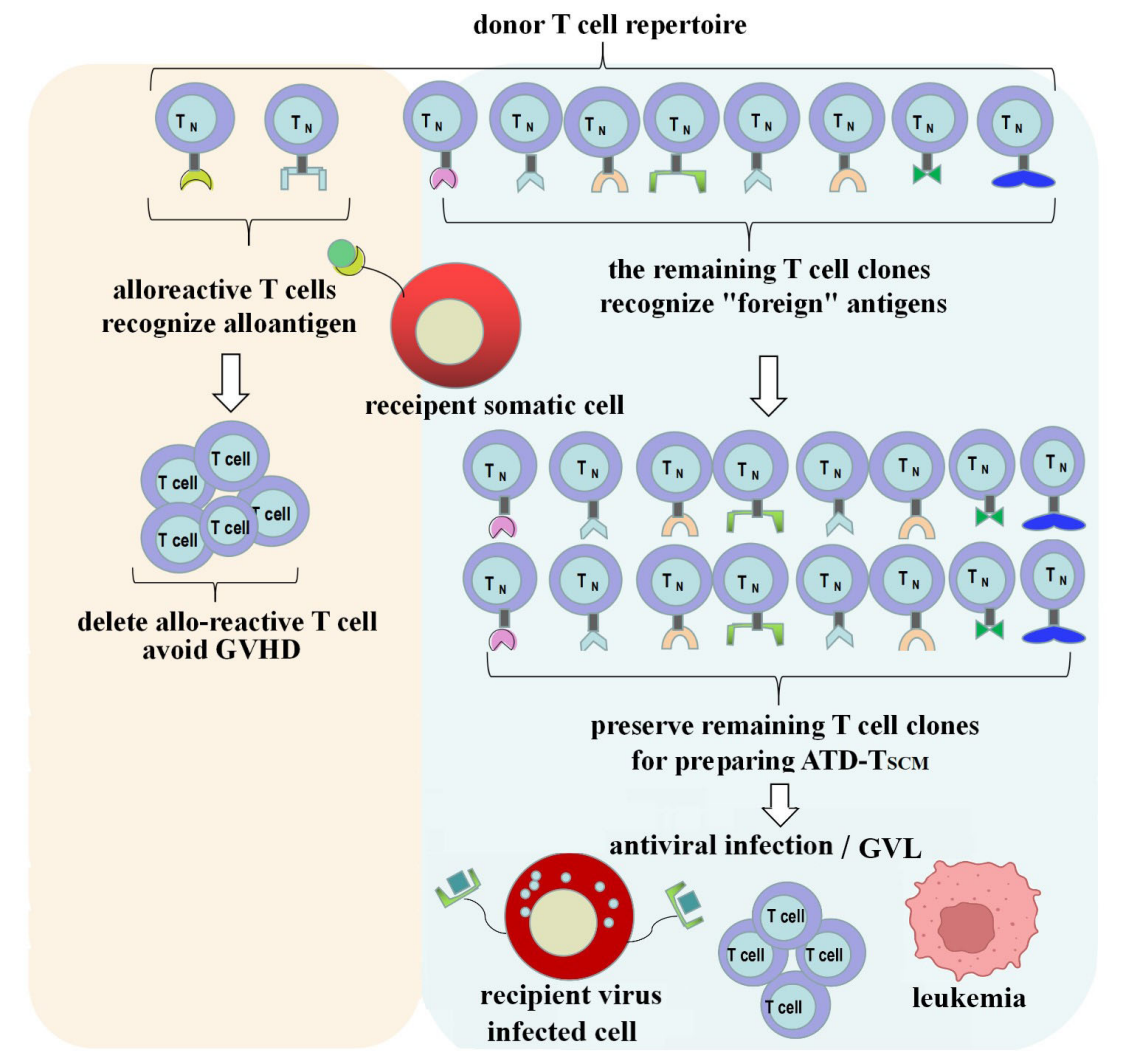
ATD-Tscm can achieve immune reconstitution while avoiding the occurrence of GVHD. Alloreactive T cell clone in the donor T cell repertoire were depleted and the vast majority of the remaining T-cell clones were used to prepare the ATD-Tscm for T-cell adoptive therapy to avoid GVHD and rebuild T cell immunity.

GVHD occurs due to T cells from the graft recognizing the recipient’s allogeneic antigens (also known as alloantigens or tissue antigens), which triggers an immune attack and leads to a rejection response (19). The T cells in the donor’s T cell repertoire that recognize these allogeneic antigens are referred to as allo-reactive T cells. They can directly recognize allogeneic pMHC and generate a response, which forms the basis for the development of GVHD, and their recognition is pMHC-specific. The precursor frequency of allo-reactive T cells is estimated to be about 0.01 to 0.1 percent of the T cell repertoire (22). The vast majority of the remaining T cell clones in the T cell repertoire will recognize “foreign” antigens, which is the foundation for immune reconstitution. Therefore, if T cell clones in the graft that target the recipient’s alloantigens can be depleted while preserving T cell clones that recognize “foreign” antigens, it is anticipated that GVHD can be avoided during the process of immune reconstitution (**Figure 8**).

T cells, after maturing in the thymus, migrate to peripheral immune organs and tissues, where they are known as naïve T cells (Tn). Upon antigen stimulation, the specific T cells undergo clonal proliferation and differentiate into various T cell subsets, such as central memory T cells (Tcm), effector memory T cells (Tem), and effector T cells (Tef), which then produce an immune response. T memory stem cells (Tscm) are a subset of T cells that possess characteristics of both memory T cells and stem cells (10–12). During the differentiation of Tn into various T cell subsets, Tscm are the earliest to differentiate. Their memory T cell characteristics are manifested by a recall response or reactivity upon re-stimulation with antigens, typically showing rapid differentiation into effector cells and mounting an immune response (11, 12, 33). Their stem cell characteristics are shown by their ability to self-renew and differentiate, which gives them good engraftment potential. In the absence of antigen stimulation, the lifespan of naive lymphocytes is generally only a few month (13–15), while Tscm have strong self-renewal and differentiation capabilities within the body, and their survival and self-renewal do not require antigen stimulation, allowing them to survive for several decades after infusion (16–19). Therefore, Tscm can serve as seed cells for allogeneic T cell immune reconstitution.

The differentiation of T cells is a series of continuous processes. Naive T cells (Tn cells) express CD45RA and also express lymphocyte homing molecules such as L-selectin (CD62L), CCR7, and co-stimulatory receptors CD27 and CD28. These membrane proteins facilitate the smooth entry of T cells into secondary lymphoid organs, allowing them to interact with antigen-presenting cells (APCs). Under the influence of APCs presenting antigens (activation signal 1), co-stimulatory molecules (activation signal 2), and cytokines (activation signal 3), Tn cells enter an activated state. After proliferation, CD62L, CCR7, and CD45RA are gradually downregulated. With the continuous change in phenotype, they differentiate into various T cell subpopulations (34, 35). Tscm cells are phenotypically similar to Tn cells but also express molecules not expressed by Tn cells, such as CD95 and CD122 (IL-2Rβ). Therefore, human Tscm are often identified phenotypically by the markers CD45RA+, CD62L+, CD95+, CCR7+, CD27+, CD28+, and CD122+/CXCR3+. Mouse Tscm are typically identified phenotypically by markers such as CD44^low^, CD62L^high^, stem cell antigen-1 (Sca-1)^high^, and CD122^high^/CXCR3^high^. And the Tscm subset with high expression of CD122 demonstrates greater proliferation, greater multipotency, and enhanced polyfunctionality, with higher frequencies of cells producing triple positive cytokines (TNF-α, IL-2, IFN-γ) upon exposure to recall antigens (36, 37).

In the research conducted by K. Jimbo and colleagues, it was observed that there was a significant increase in CD4+ Tscm (T stem cells) in patients with chronic graft-versus-host disease (cGVHD) following allogeneic hematopoietic cell transplantation. They also suggested that the levels of ICOS in Tscm are associated with cGVHD (38). Britt E. Anderson, et al. analyzed the reasons why Tem are less likely to cause GVHD in MHC-matched and MHC-mismatched mouse strain pairings, suggesting that Tem may have a more restricted TCR repertoire that reduces the frequency of alloreactive T cells below the threshold necessary to induce GVHD (39). However, Tn has a frequency of alloreactive T cells as high as 10%, therefore, Tn and Tscm are more likely to induce GVHD compared to Tem. The method of depleting Tn from the graft was used to prevent the occurrence of GVHD in the study of Marie Bleakley and colleagues. Tn contains many alloreactive T cells, and their depletion can indeed prevent GVHD (40). However, since Tn also contains a large number of different T cell clones that target foreign antigens, their removal would significantly weaken the immune response capability of the adoptively transferred cells. In this study, We initially stimulated nearly all alloreactive T cells in Tn to become activated and proliferate through a co-culture method. Then, we completely eliminated this group of cells using flow cytometry sorting, which eradicated the basis for the development of GVHD. Building on this, we prepared the remaining cells into ATD-Tscm cells by referencing the methods for preparing Tscm cells by Nicoletta Cieri, et al (28) as well as L. Gattinoni and colleagues (10), with the aim of achieving immune reconstitution without causing GVHD.

We utilizes two groups of mice with different MHCs, serving as recipients and donors, respectively, to prepare Tscm that are unresponsive to the recipient’s allogeneic antigens, specifically Tscm depleted of alloreactive T cell clones (ATD-Tscm). Initially, lymphocytes from the recipient mouse are used as stimulators to co-culture with lymphocytes from the donor mouse, activating and proliferating T cell clones in the donor’s T cell repertoire that are reactive to the recipient’s allogeneic antigens. By leveraging the principle of CFSE fluorescence dilution in proliferating cells and employing flow cytometry cell sorting, these alloreactive T cell clones are removed from the donor’s T cell repertoire, preserving the clones that recognize “foreign” antigens and inducing specific unresponsiveness to the recipient’s allogeneic antigens, thereby achieving immune tolerance. Subsequently, the preserved T cell clones are collected and subjected to three steps: non-specific stimulation with CD3/CD28 antibodies, differentiation inhibition with inhibitors, and cytokine expansion, to prepare Tscm that are unresponsive to the recipient’s allogeneic antigens, namely ATD-Tscm. After confirming *in vitro* that the prepared ATD-Tscm possess stem cell characteristics, memory T cell characteristics, and features of the T cell repertoire. Concurrently, the antiviral and anti-leukemic effects are observed. They are adoptively transferred into the recipient mice with the aim of long-term engraftment, differentiation into various T cell subsets, and achieving immune reconstitution without the occurrence of GVHD. Tscm, as a unique subset of memory T cells, has been reported to preferentially reside in the bone marrow after adoptive transfer (41). We observed that the prepared ATD-Tscm cells migrate to secondary lymphoid organs, such as the bone marrow and spleen, in the recipient mice, demonstrating long-term survival potential of at least 42 days post-transfer in our model. Importantly, the number of ATD-Tscm cells observed in blood samples was consistent throughout the study, suggesting that the transferred ATD-Tscm cells possess the ability to self-renew within the body. The engraftment and long-term persistence of ATD-Tscm cells within the host’s body.

Taken together, this study helps to further understand the allograft immune response and provides an experimental basis for immune reconstitution. This study helps to further understand the allograft immune response and provides an experimental basis for immune reconstitution. However, there are still many problems to be solved before clinical application, such as the safety of CFSE staining and the indications for ATD-Tscm cell adoption.

## 5 Conclusion

Allogeneic T cell adoptive therapy can directly replenish the number of T cells, rapidly reconstruct T cell immune function, and thus exert anti-infective and anti-tumor effects. However, the poor engraftment of the adoptively transferred T cells and the occurrence of GVHD limit its application. This study utilizes the principle of allogeneic rejection reactions to deplete the donor’s T cell repertoire of allo-reactive T cell clones. Based on the characteristics of Tscm that possess both memory T cell and stem cell features, we prepare Tscm depleted of allo-reactive T cells for T cell adoptive therapy. This approach aims to avoid GVHD and to reconstruct T cell immunity.

## Data availability statement

The raw data supporting the conclusions of this article will be made available by the authors, without undue reservation.

## Ethics statement

The animal study was approved by Animal Care Committee of Xinxiang Medical University. The study was conducted in accordance with the local legislation and institutional requirements.

## Author contributions

LG: Funding acquisition, Methodology, Writing – original draft. YS: Investigation, Methodology, Writing – review & editing. YLS: Formal analysis, Software, Writing – review & editing. QY: Data curation, Writing – review & editing, Investigation. ZH: Data curation, Writing – review & editing. YL: Supervision, Validation, Writing – review & editing. TH: Conceptualization, Funding acquisition, Writing – review & editing.
