# Supplementary material for: A strategy to reconstitute immunity without GVHD via adoptive allogeneic Tscm therapy
Source: Front Immunol. 2024 Jul 5;15:1367609. doi: 10.3389/fimmu.2024.1367609 (PMC11259968; doi:10.3389/fimmu.2024.1367609)
Supplement: Supplementary file 1 [file DataSheet_1.pdf]

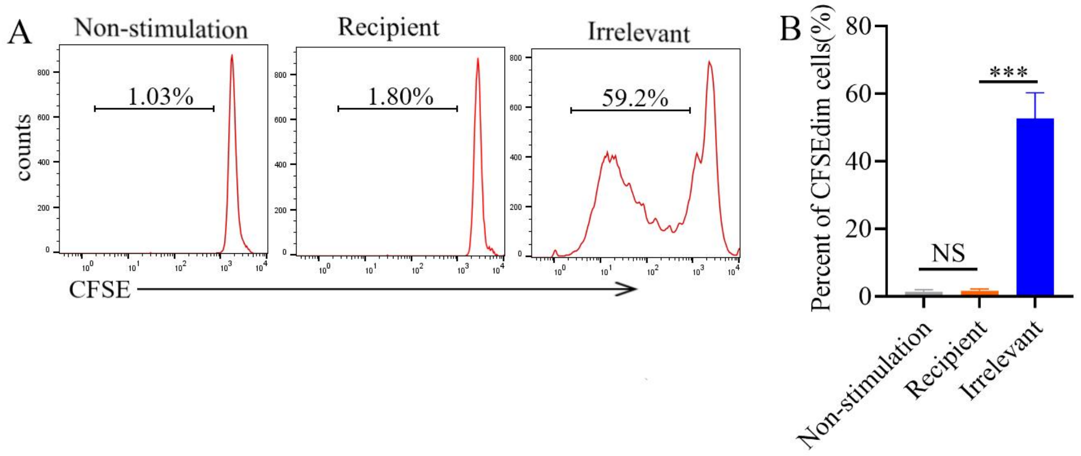

Supplementary Figure S1. Alloreactive T cells were completely removed by coculture and sorting. Sorted CFSEbright lymphocytes were cocultured with lymphocytes (both inactivated) from recipient and irrelevant mice for one week. CFSE fluorescence was detected by flow cytometry. A. Representative FCM plots. B. The frequencies of CFSEdim cells are shown. The data are presented as the means ± SDs of six individual experiments.
